# Strengthening patient safety through biosafety and infection prevention measures during COVID-19: a policy and practice review

**DOI:** 10.3389/frhs.2026.1914377

**Published:** 2026-09-24

**Authors:** Zixuan Gao, Shiyu Wang, Yaqin Zhang, Shuojie Wang, Dianya Qiu, Linmei Yao, Tianyu Ma, Weihua Cao, Wen Deng, Xin Wei, Minghui Li, Yao Xie

**Affiliations:** 1 Department of Hepatology Division 2, Beijing Ditan Hospital, Capital Medical University, Beijing, China; 2 Department of Hepatology Division 2, Peking University Ditan Teaching Hospital, Beijing, China

**Keywords:** biosafety, COVID-19, health-system resilience, infection prevention and control, patient safety

## Abstract

The coronavirus disease 2019 (COVID-19) pandemic caused by severe acute respiratory syndrome coronavirus 2 (SARS-CoV-2) placed substantial pressure on global healthcare systems, biosafety management, and the delivery of safe clinical services. As a critical component of infection prevention and control (IPC), biosafety contributes not only to limiting pathogen exposure but also to protecting healthcare workers, maintaining reliable diagnostic pathways, and supporting patient safety and health-service continuity. This Policy and Practice Review presents a structured narrative synthesis of a sample of literature published primarily between January 2020 and August 2026, supplemented by selected earlier foundational studies and relevant public-health guidance. The review focuses on respiratory exposure control, appropriate use and oversight of personal protective equipment (PPE), airway-management safety, emergency respirator decontamination and reuse strategies including ultraviolet germicidal irradiation (UVGI), specimen collection and transport, laboratory biosafety, and emerging diagnostic technologies. The evidence indicates that safe practice requires layered, risk-based measures adapted to the exposure context and specific clinical or laboratory activity. During severe PPE shortages, UVGI was investigated as a contingency strategy for respirator decontamination, but microbial inactivation, preservation of filtration performance, and maintenance of fit or seal integrity represent distinct outcomes and require separate validation. Safe specimen pathways similarly depend on validated collection, transport, storage, and laboratory procedures that preserve both worker safety and diagnostic reliability. Emerging CRISPR- and nanomaterial-enabled technologies may improve diagnostic accessibility or turnaround time, but their contribution to patient safety depends on clinical validation, quality assurance, regulatory oversight, and successful integration into healthcare workflows. Overall, lessons from the COVID-19 pandemic support a multilayered, activity-specific approach to biosafety that integrates occupational protection, diagnostic safety, patient safety, and health-system resilience.

## 1 Introduction

The coronavirus disease 2019 (COVID-19) pandemic, caused by severe acute respiratory syndrome coronavirus 2 (SARS-CoV-2), posed an unprecedented challenge to global health systems, healthcare delivery, and patient safety (1–3). COVID-19 is an acute respiratory infectious disease with potential systemic manifestations, commonly presenting with symptoms such as fever, cough, and dyspnea (4). The high transmissibility of SARS-CoV-2, its evolving virological and epidemiological landscape, and the profound disruption caused to healthcare systems highlighted the need for adaptable biosafety and infection prevention and control (IPC) strategies (1–3).

In healthcare settings, biosafety encompasses the policies, practices, engineering controls, personal protective measures, and organizational systems used to minimize biological risks to patients, healthcare workers, laboratory personnel, and the surrounding environment (5, 6). In this review, biosafety refers specifically to measures intended to reduce accidental biological exposure and infection risks during healthcare and laboratory activities, whereas IPC refers to the broader set of clinical and organizational measures used to prevent transmission of infectious agents within healthcare settings. Occupational safety is discussed where it directly intersects with biological exposure among healthcare workers. Biosecurity, which principally concerns the prevention of loss, theft, misuse, diversion, or intentional release of biological materials, is outside the primary scope of this review. The term bioprotection is therefore not used as a separate conceptual category. During the COVID-19 pandemic, biosafety and IPC became closely interconnected across clinical care, specimen management, diagnostic laboratories, and occupational health (2, 7, 8). From a patient safety perspective, failures in biosafety and IPC can extend beyond occupational exposure. Healthcare-associated transmission may directly harm vulnerable patients (2); inadequate specimen collection or handling may contribute to diagnostic error or delay (7, 9); laboratory disruptions may compromise continuity of care (8); and shortages or inappropriate use of personal protective equipment (PPE) may affect both workforce availability and the safe delivery of clinical services (10, 11). Biosafety measures should therefore be understood as part of a multilayered patient safety system rather than solely as occupational or laboratory protection (8). Against this background, this Policy and Practice Review synthesizes evidence and practical lessons from the COVID-19 pandemic across respiratory transmission control, PPE, airway management, specimen pathways, laboratory biosafety, and emerging diagnostic technologies. Particular emphasis is placed on how these measures influence patient safety, healthcare worker protection, diagnostic reliability, and continuity of health services (12). The review also considers how lessons from COVID-19 can inform risk-based biosafety strategies and strengthen health-system resilience in future respiratory infectious disease emergencies. These interconnected domains are summarized in a multilayered biosafety framework centered on patient safety and health-system resilience (Figure 1).

**Figure 1:**
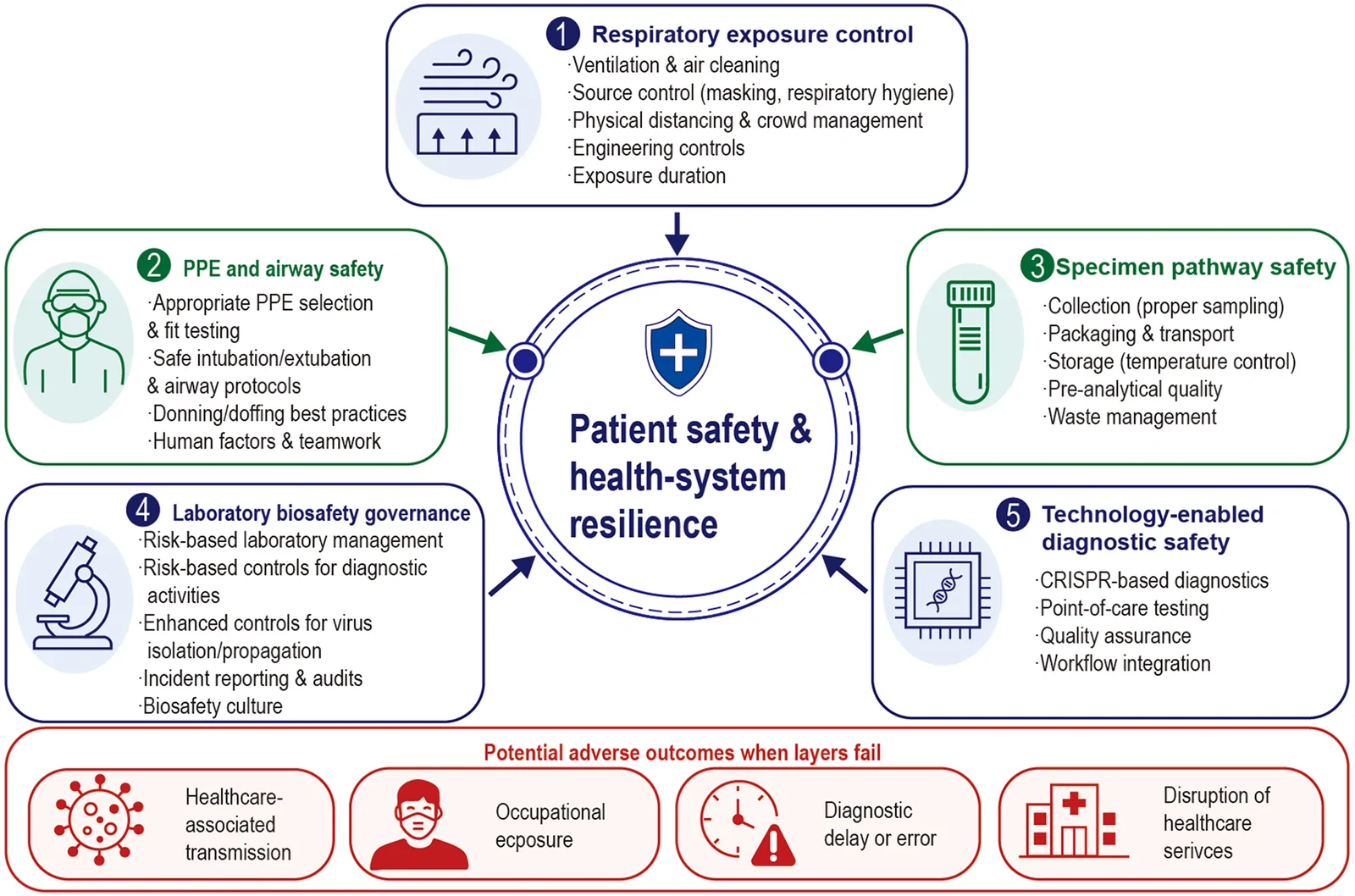
Multilayered biosafety framework for patient safety and health-system resilience. Five complementary layers—respiratory exposure control, PPE and airway safety, specimen pathway safety, laboratory biosafety governance, and technology-enabled diagnostic safety—work together to reduce occupational exposure, healthcare-associated transmission, diagnostic errors, and service disruption. The framework was developed by the authors for this review and is not adapted from a previously published model.

## 2 Methods

This Policy and Practice Review used a structured narrative approach informed by the SANRA framework (13). PubMed and Web of Science were searched for literature published from January 2020 to August 25, 2026. Google Scholar was used only as a supplementary tool to cross-check potentially relevant publications and guidance identified through the primary database searches and reference lists; it was not used as an independent source for formal study identification or inclusion. No additional publications were included solely on the basis of Google Scholar searching. No language restrictions were applied. Search terms included combinations of “COVID-19,” “SARS-CoV-2,” “biosafety,” “infection prevention and control,” “patient safety,” “healthcare worker safety,” “personal protective equipment,” “respirator,” “infectious respiratory particles,” “airborne transmission,” “airway management,” “ultraviolet germicidal irradiation,” “specimen collection,” “specimen transport,” “laboratory biosafety,” “point-of-care testing,” “CRISPR diagnostics,” and “nanomaterial biosensors.” These terms were combined using Boolean operators (AND/OR), with COVID-19/SARS-CoV-2 terms linked to topic-specific terms, and the search syntax was adapted separately for PubMed and Web of Science.

The primary database searches retrieved 768 records from PubMed and 624 from Web of Science. After removal of duplicates across these two databases, 986 records remained for screening. Four authors participated in literature screening, evidence extraction, and thematic synthesis. Potentially relevant publications were assessed against the predefined scope of the review, and uncertain cases were resolved through discussion. Extracted information included publication year, publication type, biosafety domain, healthcare or laboratory setting, intervention or practice examined, relevant safety or service outcome, and major limitations.

Peer-reviewed original studies, systematic reviews and meta-analyses, narrative or scoping reviews, consensus statements, professional recommendations, and guidance documents were considered when relevant to biosafety, IPC, patient safety, diagnostic safety, or health-service delivery. Publications focused mainly on basic molecular mechanisms or experimental biotechnology without a clear healthcare or biosafety application were excluded. Selected pre-2020 publications were retained when they provided foundational evidence relevant to PPE, airway management, UVGI, laboratory containment, or infection-control practice.

Evidence was synthesized narratively and organized into the predefined thematic domains reflected in the structure of this review. No formal qualitative coding framework or quantitative meta-analysis was applied. When findings or recommendations differed, greater weight was given to current public-health or professional guidance, recent systematic reviews or meta-analyses, and clinically applicable implementation studies. The final synthesis included 102 publications and guidance documents: 41 (40.2%) original studies, 14 (13.7%) systematic reviews and meta-analyses, 33 (32.4%) narrative, scoping, or other review articles, 6 (5.9%) guidelines, consensus statements, or professional/public-health recommendations, and 8 (7.8%) methodological, commentary, perspective, or other foundational publications. No formal risk-of-bias assessment or certainty-of-evidence grading was performed because this was a structured narrative rather than a systematic review. The complete search strategies for PubMed and Web of Science are provided in Supplementary Table S1.

## 3 Personal protection and biosafety as patient safety interventions

During the COVID-19 pandemic, PPE was a central component of IPC strategies used to protect healthcare workers and reduce healthcare-associated transmission (14, 15). Maintaining an adequately protected workforce also supported patient safety by preserving healthcare capacity and continuity of care during periods of intense service demand (14). The type and level of PPE required depend on the anticipated exposure and local risk assessment and may include respirators, eye protection, gloves, and protective gowns (15). These supplies not only serve as essential components in preventing the transmission of SARS-CoV-2 but also enable medical personnel to provide effective patient care while ensuring their own safety.

### 3.1 Intensive care unit biosafety and personal protective measures during the COVID-19 pandemic

Current international terminology recognizes that individuals infected with SARS-CoV-2 release infectious respiratory particles (IRPs) across a continuous spectrum of sizes during breathing, speaking, coughing, sneezing, and other respiratory activities (16, 17). This terminology describes the physical continuum of respiratory particles but does not, by itself, establish the effectiveness of any particular infection-control intervention. Transmission risk reflects the interaction of particle generation and dispersion, proximity to the source, exposure intensity and duration, ventilation, source control, respiratory protection, host susceptibility, and environmental conditions (16, 17). Accordingly, rigid particle-size cut-offs should not be interpreted as universal clinical boundaries for transmission or prevention. In intensive care units (ICUs), procedures involving airway manipulation, including endotracheal intubation, tracheostomy, and cardiopulmonary resuscitation, may generate or disperse respiratory particles and can increase occupational exposure when appropriate controls are absent (18, 19). Although ICU medical staff are generally familiar with biosafety protocols, numerous challenges have emerged in adhering to infection control guidelines during the pandemic. These include shortages and inconsistent quality of PPE, as well as a lack of negative pressure rooms and effective air exchange systems (10, 20).

IRPs may deposit on the eyes, nose, or mouth or may be inhaled into the respiratory tract. Smaller particles can remain suspended in indoor air, particularly in poorly ventilated environments, while exposure risk generally increases with closer proximity, higher source emission, and longer duration of contact. These mechanisms are not mutually exclusive and support a layered approach to infection prevention (21). The probability of transmission is influenced by multiple interacting determinants, including infectious dose, intensity and duration of exposure, route of exposure, ventilation, source-control measures, host susceptibility, and environmental conditions (22). Medical procedures should also be considered according to whether they generate additional respiratory particles or predominantly disperse, redirect, or increase exposure to particles already released by the patient. Appropriately selected and fitted PPE provides an important barrier that reduces occupational exposure to infectious respiratory particles (15). Eye protection may be provided by appropriately designed goggles or face shields; a full-facepiece elastomeric respirator may also provide eye protection when used in accordance with its approved configuration, whereas a half-facepiece elastomeric respirator does not provide equivalent eye coverage (20). Respiratory protection should be selected according to the anticipated exposure, applicable national or international performance standards, product quality, institutional policy, and local risk assessment rather than assuming equivalence between certification classes such as N95 and KN95 (23). Where respiratory protection is indicated, an appropriately selected respirator should be incorporated into a respiratory-protection program that includes fit testing when required, a user seal check at each use, and medical evaluation where applicable.

Reusable elastomeric respirators may serve as alternatives to disposable filtering facepiece respirators in selected healthcare settings, provided that the respirator and filters meet applicable performance requirements and that fit, cleaning, disinfection, inspection, storage, and filter replacement are addressed through validated institutional procedures (24). Full-facepiece and half-facepiece elastomeric respirators should not be treated as interchangeable because their coverage, configuration, and protective functions differ. Source-control measures, where feasible, remain complementary to rather than substitutes for appropriate respiratory protection.

Accordingly, ICU biosafety should rely on layered controls that integrate adequate ventilation, respiratory protection, source control where feasible, hand hygiene, safe clinical workflows, and appropriate environmental cleaning and disinfection (21). Ventilation, source control, and respiratory protection primarily address respiratory exposure, whereas environmental cleaning and disinfection remain important for reducing contamination of surfaces and the risk of contact-mediated transmission; these measures should not be regarded as equivalent or interchangeable (21).

### 3.2 Personal protection and biosafety in airway management during the COVID-19 pandemic

The global dissemination of SARS-CoV-2 infection has elevated the biosafety and clinical necessity of airway management into a critical issue in intensive care and emergency medicine. Airway procedures such as endotracheal intubation involve close contact with the patient's respiratory tract and may expose healthcare workers to infectious respiratory particles, emphasizing the need for appropriate respiratory protection, ventilation, and procedural planning (25). Therefore, both patient safety and provider protection must be considered in the study and practice of airway management.

Severe COVID-19 may result in hypoxemic respiratory failure requiring advanced oxygen support or invasive mechanical ventilation in selected patients (25). Procedures involving airway manipulation, including endotracheal intubation and tracheostomy, require close proximity to the respiratory tract and may increase exposure to infectious respiratory particles, particularly when procedures are prolonged or repeated or when ventilation and respiratory protection are inadequate. Accordingly, airway management should incorporate layered controls, including appropriate respiratory protection, ventilation, procedural planning, experienced personnel, effective teamwork, and minimization of unnecessary exposure (19, 25, 26). Although PPE is an important component of airway-related infection prevention, it should be combined with ventilation, procedural planning, source control where feasible, and minimization of avoidable exposure (15). PPE should be selected according to the anticipated route and intensity of exposure, the airway procedure being performed, applicable guidance, and local risk assessment, and should be donned and removed using standardized procedures designed to minimize contamination (19). Endotracheal intubation constitutes a key technique for securing airway function in those with COVID-19. Because repeated airway instrumentation may prolong procedural duration and increase the number of close-contact manipulations, airway management should be planned to maximize first-pass success using appropriately experienced personnel and suitable airway devices (27). However, first-pass success is primarily a procedural and clinical quality outcome; although it may reduce repeated instrumentation and exposure time, it should not be interpreted as a validated endpoint for prevention of SARS-CoV-2 transmission. Early in the pandemic, concerns were raised regarding the potential for high-flow nasal oxygen (HFNO) to increase environmental dispersion of respiratory particles (19). Subsequent evidence has supported a more risk-based approach in which the clinical benefits of HFNO are balanced against local ventilation, respiratory protection, patient characteristics, and institutional IPC protocols rather than avoiding HFNO solely because of aerosol concerns (28, 29). Extubation can provoke coughing, which may increase respiratory particle dispersion during close-contact airway management (19, 30). During extubation, strategies to minimize avoidable coughing and secretion dispersal may be considered according to the patient's clinical condition and local airway-management protocols. Pharmacological strategies such as dexmedetomidine or lidocaine have been investigated for reducing coughing during emergence and tracheal extubation; however, these interventions should be considered selectively according to clinical indications, patient characteristics, potential adverse effects, and institutional protocols rather than as routine biosafety measures (31). Evidence that these agents reduce coughing during emergence or extubation does not demonstrate that they reduce SARS-CoV-2 transmission or healthcare-associated infection, and they should therefore not be regarded as infection-control interventions in the absence of direct evidence.

The COVID-19 pandemic has presented unprecedented challenges in airway management. Ensuring first-pass success in intubation, minimizing avoidable respiratory particle dispersion and exposure, and adopting appropriate protective strategies are crucial to safeguarding both healthcare workers and patients (25). Taken together, safe airway management requires integration of clinical effectiveness with occupational and patient safety. Strategies should prioritize technically successful airway management, reduction of avoidable exposure, appropriate respiratory protection, and adherence to locally validated IPC protocols rather than relying on any single intervention (28, 29).

### 3.3 Disinfection and reuse of personal protective equipment during the COVID-19 pandemic

Severe PPE shortages during the early phases of the COVID-19 pandemic prompted healthcare systems to consider emergency decontamination and limited reuse of respirators that had originally been designed for single use (32). These approaches should be interpreted as contingency measures for supply crises rather than routine PPE practice. Ultraviolet germicidal irradiation (UVGI) was among the decontamination approaches investigated as an emergency option for filtering facepiece respirators (FFRs), particularly during periods of severe supply constraint (33, 34).

UVGI technology inactivates pathogens by using ultraviolet-C (UV-C) radiation to damage microbial DNA or RNA (35, 36). Controlled laboratory studies have shown that appropriately delivered UV-C radiation can reduce viable microorganisms or experimental surrogates on respirator materials (37). However, three distinct outcomes must be evaluated separately when considering respirator reprocessing: microbial inactivation, preservation of filtration performance, and maintenance of respirator fit or seal integrity (38, 39). Demonstration of microbial inactivation does not establish that filtration performance or fit remains acceptable, and preservation of filtration performance does not demonstrate that a repeatedly used respirator continues to achieve an adequate facial seal (38). Accordingly, laboratory evidence for one outcome should not be interpreted as establishing the overall safety of clinical reuse. Several practical limitations nevertheless remain. Some UV-C systems may require substantial equipment, validation, and operational resources, which can limit their feasibility in smaller or resource-constrained healthcare settings (39). UVGI therefore requires validated dose delivery that is sufficient for the intended microbial-reduction endpoint while remaining compatible with respirator materials and performance (40). During severe PPE shortages, some institutions investigated emergency UVGI systems using existing UV-C sources, including lamps installed within laboratory biosafety cabinets (39). Such reports demonstrate the feasibility of adapting available infrastructure under crisis conditions but should not be interpreted to mean that a biosafety cabinet containing a UV-C lamp is automatically validated for respirator reprocessing. Before such a system could be considered for use, site-specific evaluation would be required, including irradiation mapping, standardized respirator positioning, cycle validation, biological or surrogate inactivation testing, assessment of material compatibility, and evaluation of respirator fit and filtration performance. These systems should therefore be regarded as context-specific emergency approaches rather than validated universal decontamination platforms. UVGI effectiveness is highly respirator-model dependent because material composition, surface geometry, folds, straps, and shadowed regions influence the dose delivered to different parts of the respirator (38). Measurement of irradiance or dose at the chamber level does not necessarily represent the dose received by shielded or poorly exposed areas of an individual respirator. Therefore, validation should include dose mapping at relevant respirator surfaces, standardized positioning, assessment of material compatibility, cycle limits, and evaluation of both filtration performance and fit or seal integrity (40). Nevertheless, studies suggest that under appropriate UV doses, the filtration capacity and structural integrity of N95 masks can remain stable even after multiple disinfection cycles (41). These findings were generally obtained under controlled conditions and should not be interpreted as support for unrestricted or routine reuse (39).

The principal lesson from respirator decontamination during COVID-19 is therefore not that disposable respirators should routinely be reprocessed, but that healthcare systems require validated contingency plans for periods of severe supply disruption. When reuse cannot be avoided, any decontamination program should incorporate method-specific validation, clearly defined reuse limits, assessment of filtration performance and fit or seal integrity, and appropriate institutional oversight (33, 38–42). Maintaining resilient PPE supply chains remains preferable to routine reliance on respirator reprocessing.

### 3.4 Implementation of PPE and biosafety measures during the COVID-19 pandemic

As a critical measure in pandemic control, PPE plays an essential role in protecting healthcare workers from viral infection and reducing disease transmission (11, 15). Guidance and published evidence on PPE use emphasize that appropriate donning and doffing sequences, together with adequate training, can reduce the risk of self-contamination (43, 44). However, despite training in the use of protective gear, self-contamination remains frequent among health workers (45).

Appropriate PPE serves as one component of a broader hierarchy of controls for reducing occupational exposure (46). This is especially important during procedures involving close respiratory exposure, such as intubation, extubation, and cardiopulmonary resuscitation, which may increase healthcare workers' exposure to infectious respiratory particles and require appropriate IPC precautions (17). Therefore, correct use and removal of PPE are essential components of infection prevention and control (43, 44). Early observational studies reported that self-contamination occurs at notably high rates during PPE use, particularly during the doffing phase, with reported frequencies ranging from 46% to 100% (47–49). From a patient safety perspective, reducing self-contamination also lowers the potential for pathogen transfer between clinical environments, staff, and vulnerable patients. Improper donning and doffing of PPE can pose serious biosafety hazards. In practice, self-contamination commonly occurs when healthcare workers remove gloves, gowns, masks, and goggles. These issues are often associated with factors such as incorrect doffing sequences, difficulty in distinguishing between clean and contaminated surfaces, rushed operations, and insufficient training (50, 51). Studies show that even when medical staff believe they are competent in proper procedures, operational errors can still occur, leading to viral exposure (52). To reduce self-contamination risks during PPE donning and doffing, interventions such as enhanced training, supervision, and technological support have been evaluated as strategies to improve procedural performance (53–55). Simulation-based training has been used to improve healthcare workers' performance in PPE donning and doffing (54). Trained observers and real-time monitoring systems may help identify procedural deviations, provide immediate corrective feedback, and reduce reliance on continuous on-site monitoring (55). Protocols for the proper use and disposal of protective gear involve multiple steps, and their complexity increases cognitive load—especially in emergency situations (15). This heightened cognitive demand can impair operational accuracy and contribute to emotional stress, thereby elevating biosafety risks. Repeated training and simulation can reinforce procedural competence and support more consistent PPE use under demanding clinical conditions (53). Therefore, regular simulation exercises and retraining may help maintain PPE handling competence among healthcare workers.

The correct use of personal protective equipment, particularly during doffing, is essential for reducing occupational exposure among healthcare workers (43). Through strengthened training, real-time monitoring, and adherence to standardized procedures, healthcare institutions can help reduce self-contamination during PPE use, improve procedural performance during outbreaks, and strengthen frontline worker safety (50).

## 4 Sample handling and biosafety during the COVID-19 pandemic

Safe and reliable specimen pathways—from collection through transport, testing, storage, and disposal—are essential to both laboratory biosafety and patient safety (6). Pre-analytical errors can contribute to false-negative or otherwise unreliable results, repeat sampling, diagnostic delays, inappropriate isolation decisions, and avoidable exposure of patients or healthcare workers (56). Table 1 presents a risk assessment framework for the collection and movement of respiratory specimens (e.g., sputum).

**Table 1:** Illustrative risk assessment for collection and transport of respiratory specimens.

Process	Risk(s) identified	Approach(es) to mitigation	Potential patient-safety consequence
Specimen collection	Environmental contamination	Verify patient identity and preparation; apply appropriate isolation and IPC precautions; ensure hand hygiene and environmental disinfection; perform collection in an adequately ventilated area where feasible	Exposure of other patients or healthcare workers and potential healthcare-associated transmission
Specimen collection	Contamination of specimen container, paperwork, or transport bag	Disinfect the outer surface of the primary container after collection; keep secondary containment outside the contaminated area where feasible; use sealed secondary packaging according to transport requirements	Cross-contamination, handling errors, and possible compromise of specimen integrity
Specimen collection	Infection or exposure of collecting staff	Use appropriate PPE and engineering controls; ensure correct patient positioning; follow standardized specimen-collection procedures; provide staff training and clear handling instructions	Occupational infection, workforce loss, and potential onward transmission within healthcare settings
Specimen collection	Inadequate or poor-quality specimen	Use validated sampling techniques and appropriate swabs; ensure adequate anatomical sampling; repeat collection when specimen adequacy is uncertain	False-negative or invalid results, delayed diagnosis, inappropriate isolation decisions, and delayed treatment
Specimen transport	Loss of specimen in transit	Use specimen tracking and chain-of-custody procedures; clearly label and document transfers	Repeat specimen collection, diagnostic delay, and disruption of clinical decision-making
Specimen transport	Breakage or damage to the primary container	Use leak-proof primary containers and robust secondary containment; avoid inappropriate pneumatic transport for high-risk specimens; comply with applicable transport regulations	Exposure of transport personnel, environmental contamination, and interruption of diagnostic workflows
Specimen transport	Leakage or spillage	Ensure secure closures, absorbent material, and compatible secondary packaging; inspect containers before transport	Occupational exposure, specimen loss, and delayed or invalid testing
Specimen transport	Temperature excursion or prolonged delay	Use validated transport conditions and monitor time and temperature when required	Reduced specimen stability, inaccurate results, and need for repeat testing

IPC, infection prevention and control; PPE, personal protective equipment. The specific control measures should be determined by local risk assessment, specimen type, transport conditions, and applicable national or international guidance.

### 4.1 Safe collection and management of SARS-CoV-2 samples

The collection of SARS-CoV-2 specimens is fundamental to clinical diagnosis, surveillance, and infection-control decision-making (57). The quality of sample collection directly affects subsequent test results, and biosafety is particularly critical when dealing with highly infectious viruses (58).

SARS-CoV-2 is mainly spread via the respiratory pathway. Collected respiratory specimens encompass nasopharyngeal swabs (NPS), nasal swabs, oropharyngeal swabs, saliva, bronchial washings, bronchoalveolar lavage fluid (BAL), endotracheal aspirates, sputum, and samples from both the upper and lower respiratory tracts. Non-respiratory specimens include blood, feces, and other body fluids, such as cerebrospinal fluid and urine (59). Coronavirus detection has utilized multiple swab materials, including nylon flocked, rayon, polyester, and cotton swabs (58). Lower respiratory tract specimens may be appropriate in selected patients when clinically indicated and when collection can be performed safely (58). During specimen collection, operators should use PPE appropriate to the anticipated exposure and local risk assessment, which may include respiratory protection, eye protection, gloves, and protective clothing (6, 60, 61). Specimen collection and initial handling should follow an activity-specific risk assessment and appropriate IPC controls. Subsequent non-propagative diagnostic processing should be conducted using controls determined by the specific procedure, specimen characteristics, validated inactivation steps where applicable, institutional risk assessment, and relevant national or international guidance (6). During specimen collection from COVID-19 suspected or confirmed cases, operators should minimize exposure to infectious respiratory particles generated during coughing, sneezing, or airway sampling procedures (17, 21). Where feasible, collection should be performed in adequately ventilated spaces with appropriate PPE and workflow controls to reduce exposure of staff and other patients.

After collection, specimens should be processed or stored under validated conditions to preserve specimen integrity and the stability of relevant diagnostic targets (61). Swab specimens should be placed in transport media or devices appropriate to the validated assay and collection system, following manufacturer instructions and current laboratory guidance (62, 63).

### 4.2 Biosafety in the transport of SARS-CoV-2 samples

Appropriate packaging and handling procedures are required to minimize leakage, environmental contamination, and occupational exposure (6, 61). Appropriate transport conditions are also important for preserving specimen integrity and analytical reliability (61, 64). Transport personnel should follow standard precautions and institutional procedures for handling packaged biological specimens, with additional PPE used when leakage, contamination, or direct exposure is reasonably anticipated (61).

SARS-CoV-2 specimens should be transported under validated time and temperature conditions appropriate to the specimen type and intended diagnostic assay in order to preserve specimen integrity and analytical reliability (65). For respiratory swab specimens, the collection device, transport medium, specimen volume, temperature, and allowable transport time should follow the requirements of the validated diagnostic assay, manufacturer instructions, current laboratory guidance, and local procedures. VTM or sterile saline and refrigerated transport at approximately 2–8°C have been used in specific SARS-CoV-2 testing protocols (63), but these conditions should be regarded as examples rather than universal requirements for all specimen types or assays. Experimental studies have shown that phosphate-buffered saline (PBS), saline, and other media may preserve SARS-CoV-2 RNA sufficiently for molecular detection under specified conditions (62, 63). These findings are assay-, specimen-, and time-dependent and should not be interpreted as evidence that one transport medium is universally superior to another. Moreover, preservation of viral RNA for molecular detection is distinct from preservation of replication-competent infectious virus. Laboratories should therefore select transport media and conditions according to validated assay requirements and the intended downstream analysis. Sterile-container specimens, including endotracheal aspirates, BAL fluid, or sputum, can be transported without universal transport medium (UTM) (61).

For diagnostic clinical specimens subject to biological-substance transport requirements, packaging should comply with the applicable national and international classification and transport regulations, which commonly include a leak-proof primary receptacle, secondary containment with appropriate absorbent material, and protective outer packaging (6). Cultures, isolates, or other materials containing or suspected to contain replication-competent virus may be subject to different classification, labelling, documentation, packaging, and transport requirements and should not be treated as equivalent to routine diagnostic specimens.

### 4.3 Biosafety in the storage of SARS-CoV-2 samples

Appropriate specimen storage is essential for preserving sample integrity and the stability of diagnostic targets before testing or further analysis. Improper storage conditions can lead to degradation of viral nucleic acids or other diagnostic targets, thereby compromising test accuracy (65).

Storage conditions for SARS-CoV-2 specimens should be determined by specimen type, transport medium, intended assay, anticipated storage duration, and validated laboratory procedures (66). Refrigerated storage at approximately 2–8°C and frozen storage at lower temperatures have been used in different diagnostic protocols, but no single temperature-duration combination should be interpreted as universally applicable to all specimens or analytical purposes. Studies evaluating stability of SARS-CoV-2 RNA for molecular detection address a different endpoint from studies assessing recovery of replication-competent virus; therefore, RNA detectability should not be equated with preservation of infectious virus (65, 66). Laboratories should follow current assay-specific instructions and national or international guidance for storage, particularly when specimens are retained for culture, research, or other purposes beyond routine nucleic-acid testing. Sample storage should be conducted in laboratories or storage facilities that meet biosafety requirements (67). These facilities should meet applicable biosafety and storage requirements and include appropriate temperature monitoring and access controls according to the specimen type and intended use (8). Storage areas should be maintained and decontaminated in accordance with institutional biosafety procedures to reduce environmental contamination; such measures complement, but do not replace, appropriate specimen containment, ventilation where relevant, PPE, and safe handling practices. Handling and storage of potentially infectious specimens may pose occupational exposure risks to laboratory personnel. According to WHO guidelines, every sample (including feces, blood, body fluids, and swabs) should be considered potentially infectious (6). Biosafety training and awareness initiatives for laboratory personnel should be part of health and safety protocols, as well as strict protocols for specimen collection, transport, storage, and handling (68).

Adherence to validated procedures for specimen collection, transport, and storage can reduce biosafety risks while supporting the accuracy and reliability of SARS-CoV-2 testing (7).

## 5 Laboratory oversight and safety practices during the COVID-19 pandemic

### 5.1 Construction of a laboratory biosafety management system

The COVID-19 pandemic highlighted the importance of robust laboratory biosafety systems as diagnostic and research activities involving potentially infectious specimens expanded rapidly. Robust laboratory biosafety management systems are therefore essential for maintaining safe laboratory operations, reducing occupational and environmental risks, and minimizing the likelihood of laboratory-acquired infection or other biosafety incidents (69, 70). A sound biosafety management system requires comprehensive planning and deployment across multiple dimensions, including policies and regulations, management protocols, operational procedures, and personnel training, to ensure compliance with biosafety requirements at every level (70).

First, laboratories must develop their institutional frameworks in accordance with national and international biosafety regulations. Contemporary laboratory biosafety increasingly follows a risk-based approach in which control measures are matched to the characteristics of the biological agent, the laboratory activity being performed, the likelihood of exposure, and the consequences of an incident (71). WHO guidance emphasizes local risk assessment, good microbiological practice and procedure, appropriate engineering controls, personnel competency, and institutional oversight rather than relying solely on fixed containment labels. National standards may further specify local requirements (6). Laboratories must evaluate their facilities and management practices against these standards to ensure regulatory compliance. In terms of organizational structure, laboratories should establish an appropriate institutional biosafety governance structure, which may include a biosafety committee and designated personnel responsible for biosafety oversight, depending on institutional size, activities, and regulatory requirements (70). These individuals should possess not only expertise in biosafety but also crisis management capabilities to respond effectively to unexpected biosafety incidents. Furthermore, laboratory biosafety management should include both regular self-inspections and external audits (8). Periodic self-assessments help identify potential biosafety risks and facilitate timely corrective actions, while external review, where applicable, can provide an additional assessment of the effectiveness and compliance of the biosafety management system. These evaluations became particularly important during the COVID-19 response because procedural failures could increase occupational exposure, compromise laboratory continuity, or affect diagnostic reliability (72).

Personnel management is also an integral component of the biosafety system. To ensure that laboratory staff possess strong safety awareness and operational skills, systematic biosafety training must be implemented. Training should cover basic laboratory environmental requirements, standard operating procedures, and emergency response measures. Particular emphasis should be placed on targeted training for personnel handling high-risk pathogens to ensure they are proficient in protective operations and capable of responding swiftly and appropriately in emergencies (73).

### 5.2 Biosafety assurance in laboratory environment and facilities

The COVID-19 pandemic has further underscored the fundamental role of laboratory environments and facility infrastructure in ensuring biosafety. The physical setting and equipment of a laboratory must not only support daily research activities but also effectively prevent pathogen leakage and mitigate viral transmission. During the pandemic, the design of laboratory spaces, selection of appropriate facilities, and optimization of operational procedures have all proven essential to maintaining biosafety (70).

Laboratory design, zoning, and operational controls should be matched to the activities performed and the risks identified through local assessment, with biosafety levels providing a general framework for containment requirements. Facility design and environmental controls vary according to the level of containment required (74). For SARS-CoV-2, the controls required for non-propagative diagnostic activities should be determined by the specific laboratory procedure and local risk assessment rather than by the pathogen name alone (6). Factors relevant to that assessment include whether intact clinical material is handled, whether a validated inactivation step has been completed for the specific specimen-assay combination, the likelihood of aerosol generation, the concentration or volume of potentially infectious material, and whether replication-competent virus may be present. National regulations and institutional biosafety requirements remain applicable. Activities involving isolation, propagation, or manipulation of replication-competent virus require additional containment and controls determined through activity-specific risk assessment and applicable regulatory requirements (75). The level of containment should reflect the biological characteristics of the agent, the procedures performed, the concentration and volume of infectious material, and the potential for aerosol generation or accidental exposure rather than relying solely on pathogen classification (76). Poorly characterized agents or variants with uncertain biological characteristics may warrant more stringent containment until their risks are adequately characterized. The resulting combination of engineering, procedural, and personal protective controls should reflect the assessed risk of the specific activity (Table 2). Where enhanced containment is required, appropriate primary containment, access controls, environmental controls, and validated decontamination procedures should be incorporated according to the identified risks. The required combination should therefore be determined through activity-specific risk assessment. Validated decontamination technologies may also be incorporated as adjunctive controls where appropriate (75). UV-based systems can contribute to environmental decontamination in selected applications but should not substitute for validated primary containment, cleaning, and disinfection procedures (77). Waste treatment facilities are another key aspect of biosafety management (70). Laboratory waste should be managed according to validated institutional procedures and applicable regulations. Infectious or potentially contaminated waste may require appropriate decontamination, such as autoclaving or validated chemical treatment, before final disposal, depending on the assessed risk and waste type.

**Table 2:** Risk-based laboratory controls for selected SARS-CoV-2-related activities.

Laboratory activity	Risk-based containment approach	Key control measures	Relevance to patient/service safety
Routine hematology or clinical chemistry using clinical specimens	Controls determined by specimen type, procedure, validated inactivation where applicable, and institutional risk assessment	Standard precautions; safe specimen handling; closed systems where available; procedures to minimize splashes or aerosols; staff competency	Supports continuity and reliability of routine diagnostic services while limiting occupational exposure
NAAT or sequencing involving primary clinical material	Activity-specific controls based on whether potentially infectious material is handled before validated inactivation	Local risk assessment; appropriate primary containment for aerosol- or splash-generating steps; validated inactivation where applicable; trained personnel; contamination control	Supports reliable molecular diagnosis while reducing occupational exposure and cross-contamination
NAAT or sequencing performed after validated inactivation	Controls based on the residual hazards of the downstream procedure and institutional requirements	Confirmation that the inactivation procedure is validated for the specimen-assay combination; standard molecular-laboratory contamination controls	Allows safe and reliable downstream testing without assuming that all molecular workflows have identical biological risk
Point-of-care or near-patient testing	Local risk assessment based on specimen collection, test format, environment, and operator exposure	Appropriate PPE; safe specimen application and disposal; operator training; quality assurance; environmental controls appropriate to the setting	Supports rapid clinical decisions while maintaining diagnostic quality and operator safety
Virus isolation or propagation	Enhanced containment determined by activity-specific risk assessment and applicable regulations	Appropriate primary and secondary containment; restricted access; trained personnel; validated decontamination; additional engineering controls where indicated	Protects personnel and services while enabling specialized surveillance or research
Novel or incompletely characterized variants or agents	Precautionary controls proportionate to uncertainty until relevant hazards are adequately characterized	Institutional biosafety review; enhanced containment where justified; reassessment as biological information becomes available	Reduces risk arising from uncertainty while avoiding inappropriate permanent escalation of containment

NAAT, nucleic acid amplification test. The terms biosafety level, containment level, and risk group describe related but distinct concepts and should not be used interchangeably. The controls required for SARS-CoV-2-related work depend on the exact activity, specimen characteristics, validated inactivation procedures, aerosol-generating potential, presence of replication-competent virus, applicable national regulations, and institutional risk assessment. The table provides an illustrative framework rather than universal containment requirements.

PPE should be selected according to the laboratory activity and anticipated route of exposure. Depending on the risk assessment, this may include laboratory coats or gowns, gloves, eye or face protection, and respiratory protection (78). PPE should complement, rather than replace, primary containment devices, engineering controls, hand hygiene, and safe microbiological practices. Hand hygiene should be performed at appropriate points before and after laboratory work and after removal of gloves or other potentially contaminated PPE.

### 5.3 Biosafety training for laboratory personnel

Personnel competency is a central component of effective laboratory biosafety. Especially during the pandemic, the safety awareness and operational competence of laboratory staff directly influence the effectiveness of the overall biosafety management system. Laboratory personnel are responsible for implementing procedures, recognizing hazards, and responding appropriately to deviations or incidents (79, 80). Throughout the pandemic, laboratory personnel needed a clear understanding of fundamental biosafety principles and key competencies, including emergency response and appropriate use of PPE. These competencies can support risk reduction and more consistent implementation of biosafety procedures.

First, biosafety training should begin with onboarding (80). New employees should receive structured instruction in biosafety principles, laboratory procedures, transmission risks, and emergency response measures. Training content should be clear, practical, and appropriate to personnel roles and assigned laboratory activities. In laboratories handling higher-risk biological materials or activities, training should be proportionate to the specific hazards and procedures identified through local risk assessment. For example, research involving SARS-CoV-2 should include education on the virus's hazards, transmission characteristics, exposure routes, and activity-specific controls (78). Training should include appropriate donning and doffing of protective clothing, correct operation of biological safety cabinets, and response procedures for spills or exposures.

Furthermore, personnel competency should be assessed using institutionally appropriate training and evaluation procedures before staff undertake activities for which specific biosafety competencies are required (78). Emergency-response training is also an important component of biosafety education. Laboratory staff should be familiar with institutionally defined procedures for responding to incidents and limiting further exposure or contamination. For example, laboratory spills should be managed according to validated institutional standard operating procedures (SOPs), including appropriate containment, decontamination, access restriction where necessary, and incident reporting (79). Depending on the nature and severity of the incident, additional measures such as restricting access, evacuation, or notification of responsible institutional personnel may be required according to local emergency procedures. A strong biosafety culture should also encourage reporting of near misses and procedural deviations without discouraging disclosure, because learning from such events is central to both laboratory quality improvement and patient safety (79).

## 6 Technology-enabled diagnostic safety and biosafety innovation

The COVID-19 pandemic accelerated the development and deployment of diagnostic and protective technologies intended to improve speed, accessibility, and biosafety across healthcare settings (81, 82). From a health-service perspective, the value of these technologies depends not only on analytical performance but also on their ability to shorten diagnostic turnaround time, support decentralized testing, reduce avoidable exposure, integrate into clinical workflows, and maintain quality in resource-constrained settings (83). CRISPR-based diagnostics and many nanomaterial-enabled biosensors discussed in this section remain emerging technologies, with levels of clinical validation, regulatory authorization, and routine implementation varying substantially among platforms. Their relevance to patient safety should therefore be considered in terms of potential health-service benefit together with the evidence supporting analytical performance, clinical validity, operational reliability, and regulatory readiness. This integrated diagnostic pathway, from specimen collection and testing to quality assurance, clinical decision-making, and patient-safety outcomes, is summarized in Figure 2.

**Figure 2:**
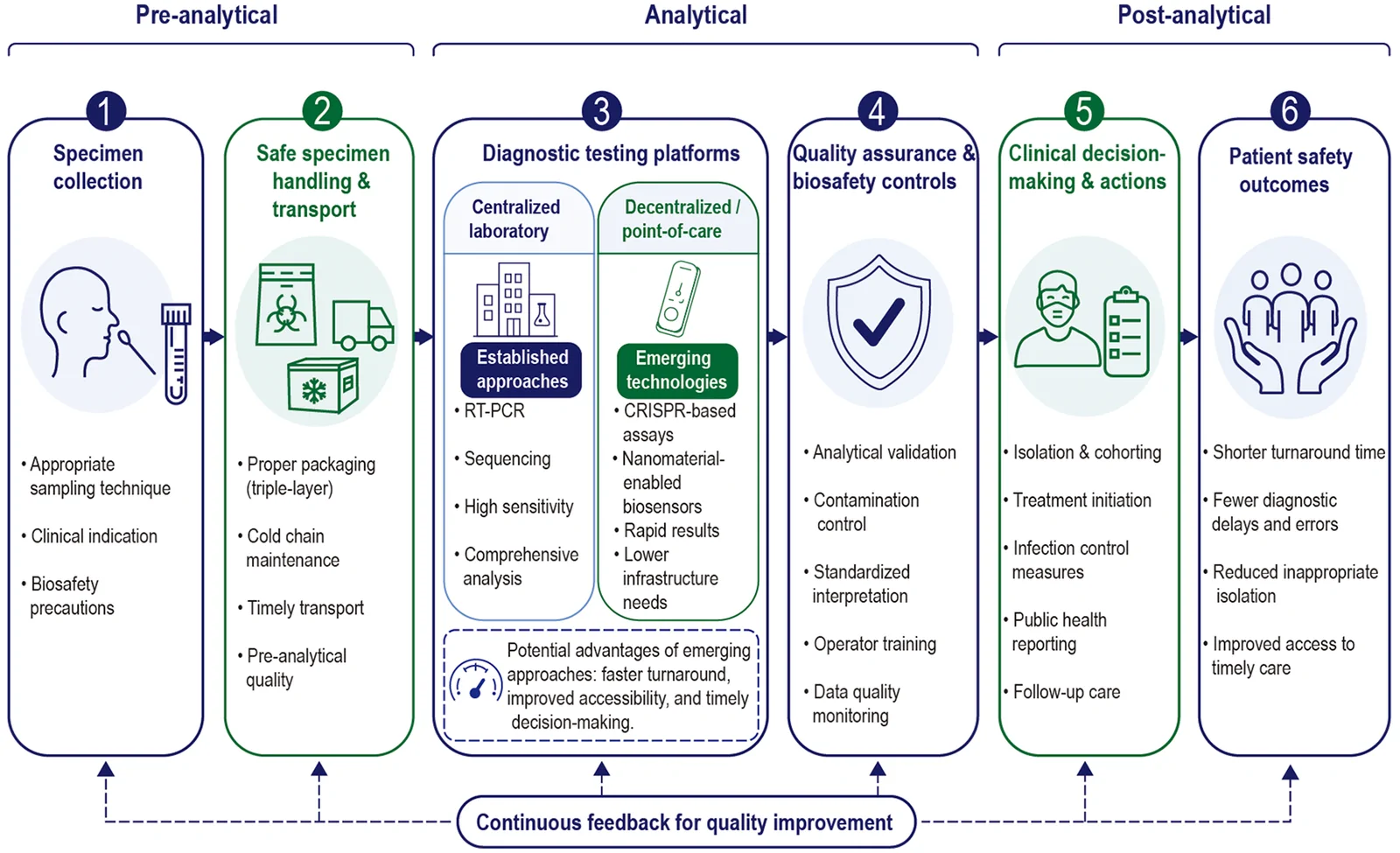
Technology-enabled diagnostic safety workflow for respiratory infectious diseases. The workflow distinguishes the pre-analytical phase (specimen collection, handling, and transport), analytical phase (diagnostic testing and quality-assurance processes), and post-analytical phase (clinical decision-making and patient-safety outcomes). Established approaches, including RT-PCR and sequencing, are differentiated from emerging technologies such as CRISPR-based assays and nanomaterial-enabled biosensors. Continuous feedback across the pathway supports quality improvement and resilient health-service delivery. The workflow was developed by the authors for this review and is not adapted from a previously published model.

### 6.1 CRISPR-based rapid and point-of-care diagnostics

Clustered regularly interspaced short palindromic repeats (CRISPR)-based diagnostic platforms attracted considerable attention during the COVID-19 pandemic because programmable nucleic-acid recognition can support sensitive detection with relatively simple readout systems (84, 85). Cas12- and Cas13-based platforms have demonstrated analytical feasibility for SARS-CoV-2 detection and may, in appropriately validated formats, offer shorter turnaround times or reduced dependence on centralized molecular laboratories (83, 86). However, analytical feasibility should be distinguished from clinical validation, regulatory authorization, and routine implementation. The extent to which these potential advantages can be realized in practice depends on specimen preparation, internal controls, contamination management, invalid-result rates, variant inclusivity, clinical sensitivity and specificity, operator training, external quality assessment, data connectivity, and applicable regulatory requirements (83).

Importantly, faster detection does not automatically translate into safer care. Clinical implementation requires rigorous analytical and clinical validation, contamination control, quality assurance, standardized interpretation, operator training, and appropriate linkage of test results to clinical decision-making (86). False-positive or false-negative results may lead to inappropriate isolation, delayed treatment, or unnecessary use of healthcare resources. Accordingly, CRISPR diagnostics should be evaluated not only by analytical sensitivity but also by their effect on diagnostic safety, turnaround time, accessibility, and workflow integration (86). Moreover, improved analytical speed or sensitivity under controlled conditions should not be equated directly with improved patient-safety outcomes.

### 6.2 Nanomaterial-enabled diagnostic and protective technologies

#### 6.2.1 Emerging nanomaterial-enabled approaches for SARS-CoV-2 diagnostics

Nanomaterial-enabled platforms were extensively investigated during the COVID-19 pandemic as emerging or proof-of-concept approaches for rapid and potentially decentralized diagnosis. Compared with conventional reverse transcription polymerase chain reaction (RT-PCR), which generally requires centralized laboratory infrastructure, trained personnel, and multiple processing steps, nanomaterial-enabled biosensors were explored as tools to improve analytical sensitivity, shorten turnaround time, and facilitate point-of-care (POC) testing (87).

For example, gold nanoparticle (AuNP)-based electrochemical and colorimetric platforms have been developed for SARS-CoV-2 detection, while graphene field-effect transistor (FET) biosensors have demonstrated rapid detection of viral targets in clinical specimens (88, 89). These platforms can convert molecular recognition events into optical or electrical signals with limited instrumentation, which may be advantageous in decentralized or resource-constrained settings (87). Most such reports represent analytical or proof-of-concept studies rather than evidence of routine clinical implementation. Important translational challenges include matrix effects in clinical specimens, reproducibility across laboratories, lot-to-lot variability, robustness across viral variants and specimen types, calibration and quality-control requirements, clinical validation, material-related biosafety, manufacturing consistency, and regulatory approval.

From a health-service perspective, however, the value of nanomaterial-enabled diagnostics depends on more than analytical sensitivity alone (90). Clinical implementation requires reliable specimen preparation, reproducibility across different viral variants and sample types, quality control, standardized interpretation, staff training, and integration with existing diagnostic pathways (83). Any future patient-safety benefit would therefore depend on whether these platforms can shorten time to reliable clinical decisions without compromising diagnostic accuracy, reproducibility, or operational quality. Emerging diagnostics provide greatest value when integrated with validated biosafety and quality-control processes.

#### 6.2.2 Nanomaterial-enabled protective materials and biosafety applications

During the COVID-19 pandemic, nanomaterials were explored as potential tools for improving the protective performance and reusability of PPE. These approaches included antiviral or self-decontaminating surface coatings, photocatalytic filter materials, and nanofiber-based membranes designed to reduce viable pathogen contamination on mask surfaces (91, 92). From a biosafety perspective, their potential value lies in combining physical filtration with additional antimicrobial or self-cleaning functions, which may reduce contamination during use, handling, and limited reuse under emergency conditions (93, 94).

The severe demand for masks and other PPE during the pandemic also stimulated interest in reusable and self-decontaminating protective materials (94). Nanomaterial-enabled fabrics and coatings were investigated as approaches to reduce surface contamination, extend functional use, and potentially decrease the environmental burden associated with repeated disposal of single-use PPE. Antiviral surface coatings have also been investigated to reduce viable pathogen contamination on protective materials through contact-mediated antimicrobial activity (95). Photocatalytic and metal-organic framework-based filter materials have similarly been explored to combine particulate filtration with antimicrobial activity, while nanofiber-based membranes have been developed to provide sustained antimicrobial activity and support reusable protective applications (91). Photoactive nanocomposite coatings have also been incorporated into mask materials to provide self-decontaminating and potentially reusable protective surfaces under controlled conditions (94).

Despite these promising experimental findings, the practical value of nanomaterial-enhanced PPE depends on factors beyond laboratory antiviral activity. Durability during repeated use, maintenance of filtration efficiency and facial fit, potential toxicity or skin irritation, manufacturing scalability, cost, environmental impact, and regulatory validation all require careful evaluation before routine clinical adoption (91). In addition, self-decontaminating materials should not be assumed to eliminate the need for standard cleaning, replacement schedules, fit testing, or established IPC procedures. Demonstration of antiviral activity on a material surface does not establish protection against inhalation of infectious respiratory particles and does not demonstrate a reduction in healthcare-worker infection rates. Such materials should not replace certified, appropriately fit-tested respiratory protective devices or established IPC measures.

Accordingly, most nanomaterial-enabled protective technologies should currently be regarded as emerging or experimental adjuncts rather than established components of routine clinical PPE practice. Their future contribution to patient and healthcare worker safety will depend on whether laboratory performance can be translated into reproducible, affordable, and regulatorily acceptable products for routine healthcare use (96).

### 6.3 Implementation considerations for health services

For emerging diagnostic and protective technologies, implementation should be assessed using health-service outcomes in addition to laboratory performance. Relevant considerations include turnaround time, diagnostic accuracy, cost, infrastructure requirements, staff training, accessibility, regulatory oversight, interoperability with existing workflows, and the consequences of erroneous results (83). Technologies that perform well under laboratory conditions may provide limited patient-safety benefit if they cannot be reliably implemented in routine clinical environments. Implementation should also consider equity, because technologies that require specialized infrastructure or high per-test costs may widen rather than reduce disparities in access to timely diagnostics (83).

Evaluation of biosafety interventions should also include measurable process and patient-safety outcomes rather than relying solely on technical performance. Relevant indicators may include healthcare-associated infection rates, documented healthcare-worker exposure events, PPE-related contamination or protocol breaches, specimen leakage or rejection, diagnostic turnaround time, invalid or rejected test results, false-positive and false-negative findings, staff absenteeism, treatment delays, and disruption of essential clinical services. These outcomes should be interpreted together with potential trade-offs. More intensive PPE requirements or increasingly complex workflows may reduce some exposure risks but can also impede communication, increase fatigue and cognitive burden, prolong clinical procedures, consume limited resources, and introduce additional opportunities for procedural error. The objective of risk-based biosafety is therefore not maximal intervention at every step, but a proportionate combination of controls that achieves meaningful protection while preserving diagnostic quality, timely care, and health-service continuity.

## 7 Perspectives

The COVID-19 pandemic demonstrated that biosafety is not an isolated technical function but an integral component of patient safety and health-system resilience (97). Effective responses depended on coordinated control of respiratory exposure, appropriate PPE, safe specimen pathways, laboratory risk management, trained personnel, and reliable diagnostic services (98, 99). Failures in any one layer could increase occupational exposure, healthcare-associated transmission, diagnostic delay, or disruption of essential clinical services (100).

A key lesson for future respiratory infectious disease emergencies is the need to move from rigid, pathogen-based rules toward adaptable risk-based approaches (6). Updated international frameworks for respiratory transmission and laboratory biosafety emphasize exposure context, activity-specific risks, ventilation, engineering controls, personnel competency, and local epidemiology (101). Such approaches allow healthcare systems to maintain safety while avoiding unnecessary measures that may consume resources without proportional benefit (97).

Several limitations of this review should be acknowledged. Because the review used a structured narrative rather than systematic-review methodology, literature retrieval was not intended to be exhaustive, and no formal risk-of-bias or certainty-of-evidence assessment was performed. The included evidence was heterogeneous in design, setting, and outcome definition, and some topics—particularly emerging diagnostic and protective technologies—remain supported primarily by experimental or proof-of-concept studies. Conclusions regarding these areas should therefore be interpreted as practice-oriented synthesis and future directions rather than definitive estimates of clinical effectiveness. Future preparedness should include resilient PPE supply chains, validated contingency procedures, laboratory surge capacity, effective ventilation strategies, competency-based workforce training, and systems for rapid communication of changing guidance (98). Emerging technologies should be incorporated only when they demonstrate practical advantages in diagnostic safety, turnaround time, accessibility, or service continuity and when appropriate quality assurance and regulatory oversight are available (102).

The enduring contribution of COVID-19 biosafety experience is thus not a single technology or protective measure, but a multilayered patient safety framework in which occupational protection, infection prevention, diagnostic quality, and health-service resilience are mutually reinforcing (100). Embedding these principles into routine healthcare systems may improve preparedness for future respiratory pathogens and other emerging biological threats.
