# Supplementary material for: Strengthening patient safety through biosafety and infection prevention measures during COVID-19: a policy and practice review
Source: Front Health Serv. 2026 Sep 24;6:1914377. doi: 10.3389/frhs.2026.1914377 (PMC13646449; doi:10.3389/frhs.2026.1914377)
Supplement: Supplementary file 1 [file Datasheet1.pdf]

Supplementary Table S1. Database-specific search strategies

Database	Search strategy
PubMed	("COVID-19" OR "SARS-CoV-2") AND ("biosafety" OR "infection prevention and control" OR "patient safety" OR "healthcare worker safety" OR "personal protective equipment" OR "respirator" OR "infectious respiratory particles" OR "airborne transmission" OR "airway management" OR "ultraviolet germicidal irradiation" OR "specimen collection" OR "specimen transport" OR "laboratory biosafety" OR "point-of-care testing" OR "CRISPR diagnostics" OR "nanomaterial biosensors")
Web of Science	TS=(("COVID-19" OR "SARS-CoV-2") AND ("biosafety" OR "infection prevention and control" OR "patient safety" OR "healthcare worker safety" OR "personal protective equipment" OR "respirator" OR "infectious respiratory particles" OR "airborne transmission" OR "airway management" OR "ultraviolet germicidal irradiation" OR "specimen collection" OR "specimen transport" OR "laboratory biosafety" OR "point-of-care testing" OR "CRISPR diagnostics" OR "nanomaterial biosensors"))

Note: The search strategies shown above represent the final Boolean structures used for database searching. Publication-date limits (January 2020 to August 25, 2026) were applied using the database-specific date filters. No language restrictions were applied. Google Scholar was used as a supplementary source to identify relevant guidance documents and additional publications.
